# Curcumin Inhibits Oxidative Stress and Apoptosis Induced by H_2_O_2_ in Bovine Adipose-Derived Stem Cells (bADSCs)

**DOI:** 10.3390/ani14233421

**Published:** 2024-11-26

**Authors:** Enhui Jiang, Xuanbo Chen, Yi Bi, Chuanying Pan, Xiangchen Li, Xianyong Lan

**Affiliations:** 1 Shaanxi Key Laboratory of Molecular Biology for Agriculture, College of Animal Science and Technology, Northwest A&F University, Yangling 712100, China; jiangenhui@yeah.net (E.J.); cxb04111@126.com (X.C.); biyi0312@163.com (Y.B.); panyu1980@126.com (C.P.); 2 College of Animal Science and Technology, Zhejiang A&F University, Hangzhou 311300, China; 3 Institute of Biological and Chemical Systems, Karlsruhe Institute of Technology Hermann-Von-Helmholtz-Platz 1, Eggenstein-Leopoldshafen, 76344 Karlsruhe, Germany

**Keywords:** curcumin, adipose-derived stem cells (ADSCs), oxidative stress, apoptosis

## Abstract

In livestock production, oxidative stress (OS) is ubiquitous, reducing animal productivity and product quality. Hence, investigating the mechanisms of oxidative stress in livestock and inhibiting oxidative stress-induced damage is crucial. Curcumin, a plant-derived bioactive compound, exhibits antioxidant and anti-apoptotic properties. Adipose-derived stem cells (ADSCs) from animal adipose tissue are easily accessible and possess multilineage differentiation potential. Therefore, this work utilized bovine ADSCs to establish an oxidative stress model and investigated the effects of curcumin on oxidative stress and apoptosis. Firstly, bovine ADSCs were isolated and cultured from fetal calf subcutaneous adipose tissue. Their surface markers were identified by immunofluorescence, confirming the expression of CD29, CD44, CD73, CD90, CD105 and Vimentin, but not CD34, indicative of mesenchymal stem/progenitor cell characteristics. Secondly, to explore the effects of curcumin on oxidative damage and apoptosis in bovine ADSCs, an oxidative stress model was induced using H_2_O_2_. CCK-8 assays showed significantly reduced cell viability and SOD activity, along with increased malondialdehyde (MDA) and reactive oxygen species (ROS) levels, indicating successful modeling. RT-qPCR further confirmed that 500 μM of H_2_O_2_ treatment for 24 h promoted apoptosis. Herein, CCK-8 assays indicated a significant reduction in cell viability at >8 μM of curcumin. Thirdly, using 4 μM and 8 μM of curcumin for pre-protection, 8 μM maintained SOD activity, reduced MDA and ROS, inhibited apoptosis-related gene changes (Bcl-2, Bax, Caspase-3), and suppressed apoptosis according to a TUNEL assay. Fourthly, curcumin’s autophagy-inducing potential was hypothesized, which was confirmed by increased LC3-II and decreased P62 expression upon co-treatment with 3-MA. 3-MA inhibited curcumin’s antioxidant and anti-apoptotic effects, suggesting that curcumin’s antioxidant and anti-apoptotic roles may involve autophagy induction. In conclusion, bovine ADSCs are abundant, easily accessible, and multipotent, making them suitable for in vitro expansion. Curcumin alleviated H_2_O_2_-induced oxidative stress in bovine ADSCs, with curcumin also inhibiting apoptosis, likely through autophagy induction. This study validates the protective role of curcumin in bovine ADSCs, with potential applications in livestock production.

## Simple Summary

In livestock farming, oxidative stress is common and hurts animal growth and product quality. Curcumin, a natural compound, can help fight oxidative stress and cell death. This study used cells from cow fat to create an oxidative stress model and tested curcumin’s effects. The results show that curcumin reduces oxidative stress and cell death in these cells, possibly by triggering a cellular cleanup process called autophagy. The study suggests that curcumin could be useful in protecting livestock from oxidative stress, improving their health and production.

## 1. Introduction

In intensive farming and high-yield production environments, cattle always suffer from oxidative stress. Early studies affirmed the positive effects of oxidative stress because certain antioxidants could reduce the severity of some diseases [1]. However, recent studies have indicated that oxidative stress is a significant underlying factor in metabolic disorders, which decrease cattle’s production efficiency and increase their susceptibility to diseases [2]. Therefore, exploring the mechanisms of oxidative stress and ways to inhibit oxidative stress injury in cattle is crucial.

Adipose-derived stem cells (ADSCs), a subtype of mesenchymal stem/progenitor cells residing within adipose tissue, have emerged as premier research materials owing to their vast availability and remarkable capacity for multi-directional differentiation [3], facilitating insights into animal physiological processes. Furthermore, stem cells, including ADSCs, are vital for biological processes such as tissue injury repair, self-renewal, and immune modulation, underlining their pivotal role in organismal functioning [4]. In terms of characteristics, ADSCs exhibit similarities to other stem cells, particularly bone marrow stem cells, in possessing self-renewal abilities and multipotent differentiation potential, etc. Additionally, ADSCs are notably more abundant in number compared to stem cells sourced from other origins [5].

Consequently, mitigating oxidative stress in ADSCs holds paramount importance for maintaining the homeostatic equilibrium and overall health of domestic animals. This study commences with the isolation and identification of bovine ADSCs, leveraging surface antigen detection and multi-directional differentiation induction as key verification tools, thereby establishing a foundation for subsequent investigations into oxidative stress and apoptosis mechanisms.

In recent, increasing attention has been paid to the role of natural herbs in disease remediation. Among these, curcumin is widely used due to its distinguished functions. Curcumin, a primary curcuminoid component of turmeric, belongs to the ginger family. Initially, it was used as a drug for various health problems, such as cancers, wounds, skin diseases, burns, etc. [6,7]. Furthermore, curcumin exhibits outstanding immunomodulatory, antitumor and antioxidant activities [8,9]. Curcumin is recognized as a powerful antioxidant. However, high doses of curcumin can exacerbate oxidative stress, causing bodily damage. Under certain conditions and doses, curcumin can eliminate reactive oxygen species, induce antioxidant responses, and exert antioxidant activity, restoring balance [10]. Despite its potential benefits for animal health, curcumin’s poor solubility and relative instability limit its clinical application. Recent research has shown that packaging curcumin with nanoparticles can increase its stability in biological systems and improve its clinical efficacy [11,12,13]. Studying the antioxidant effects of curcumin on cells has significant clinical implications for the treatment of various neurological diseases and cancers.

Autophagy is a self-protective mechanism of the body that maintains homeostasis by transporting damaged proteins and organelles to lysosomes for degradation [14]. Autophagic flux can be stimulated by various forms of cellular stress, including nutrient deprivation, hypoxia, toxins, DNA damage, and intracellular pathogens. Studies have shown that the occurrence of multiple diseases is closely related to the imbalance of autophagy levels in the body, such as neurological diseases, aging, osteoarthritis, and metabolic diseases [15]. These diseases are often accompanied by local inflammatory responses, making antibacterial and anti-inflammatory treatments crucial.

Furthermore, autophagy and oxidative stress are two vital physiological processes within the organism. Their interaction is evident under various pathological conditions. Specifically, oxidative stress causes cellular damage, and in response, autophagy, induced by oxidative stress, can eliminate proteins damaged by ROS and other factors [16]. Importantly, studies have demonstrated that autophagy regulates oxidative stress through multiple mechanisms. Among these, the most prevalent pathway involves the delivery of oxidative stress-related proteins to autophagosomes for degradation by P62, thereby mitigating oxidative damage [17,18].

The ability of curcumin to regulate cellular autophagy has been confirmed. In studies on liver fibrosis, curcumin has been found to enhance autophagy levels in hepatocytes, inhibit epithelial–mesenchymal transition, and consequently suppress the occurrence and progression of liver fibrosis, demonstrating potent anti-fibrotic effects both in vitro and in vivo, with the selective protection of hepatocytes against various liver injuries [19]. Recent research has revealed that curcumin can effectively prevent hepatic steatosis in experimental non-alcoholic fatty liver disease rats by activating autophagy to regulate inflammatory responses and oxidative stress, offering a promising therapeutic direction for diseases such as liver cancer and fibrosis [20]. Although curcumin exhibits promising therapeutic effects for multiple diseases, further studies are needed to establish its dosing standards and specific regulatory mechanisms.

Curcumin is regarded as a cellular protectant that can regulate metabolic functions. For instance, a certain concentration of curcumin can inhibit the viability of preadipocytes, leading to apoptosis and reducing lipid accumulation in adipocytes, ultimately improving obesity in the body. This offers a novel approach for the treatment of metabolic syndromes [21]. Curcumin modulates apoptosis through various pathways. In studies on pancreatic β-cells, researchers found that curcumin can regulate palmitate-induced apoptosis via the PI3K-Akt-FoxO1 signaling pathway [22]. Additionally, when alveolar macrophages are treated with LPS, it triggers an acute inflammatory response, further leading to apoptosis in these cells. However, curcumin mitigates LPS-induced inflammatory responses and apoptosis in macrophages by downregulating miR-132 expression and upregulating HMGB1 expression [23].

In summary, curcumin exerts significant regulatory effects on autophagy, apoptosis, and oxidative stress. It is crucial to investigate the pharmacological effects, mechanisms of action, and clinical applications of curcumin. This article focuses on studying the regulatory roles of curcumin in apoptosis, autophagy, and its antioxidant capacity. Curcumin not only treats and prevents diseases but is also frequently applied in livestock and poultry production [24]. For instance, it can enhance the antioxidant capacity of dairy products, extending their shelf life, and effectively reduce stress responses in livestock and poultry [25]. Aflatoxin B1 (AFB1) is highly toxic to various animal species, particularly causing liver diseases. Studies have shown that curcumin significantly alleviates AFB1-induced liver toxicity and oxidative stress levels in AFB1-induced liver injury models in mice [26]. However, the low systemic bioavailability of oral curcumin limits its potential application prospects. Therefore, many research teams are committed to improving the bioavailability of curcumin [27].

In summary, besides its positive effects on treating various inflammatory conditions, curcumin’s potent antioxidant properties also yield favorable outcomes in livestock and poultry production, enhancing the economic efficiency of the animal husbandry industry to a certain extent. Researching the mechanisms of curcumin’s actions holds significant importance for livestock and poultry production.

## 2. Materials and Methods

All experimental procedures were approved by the Institutional Animal Care and Use Committee (IACUC) of Northwest A&F University (NWAFU, Yanglin, China) (Approval NO. DK2022032). The use of experimental animals was approved by the local animal welfare laws, guidelines, and policies.

### 2.1. Materials

Curcumin (C1386, Sigma, St. Louis, MI, USA) was dissolved in DMSO solution for treating cells. The final concentrations of the cell viability tests were 0 μM, 2 μM, 4 μM, 8 μM, 16 μM, and 32 μM. Final concentrations of 4 μM and 8 μM were used in other cell experiments. Cells were treated with various concentrations of curcumin dissolved in DMSO, with the final DMSO concentration kept consistent across all groups. The working concentration of DMSO in the above cell experiments should be controlled below 0.1%.

3-MA (M9281, Sigma, USA) was first diluted with PBS and then dissolved at 50 °C for 30 min before being added. The 3-MA was then diluted to 10 mM with basic medium and used for cell treatment.

### 2.2. Isolation and Culture of Cattle Adipose Derived Stem Cells (Cattle ADSCs)

Subcutaneous adipose tissue was isolated from fetal cattle at 5 to 6 months and rinsed over 3 to 5 times in PBS (1001002, Gibco, Waltham, MA, USA). The subcutaneous adipose tissue was cut into pieces and digested with 0.1% collagenase I (17100-017, Gibco, USA) for 1 h. Then, the enzymatic activity was neutralzed with equal DMEM/F12 medium with 10% fetal bovine serum (FBS) (P30-2602, PAN, Munich, Germany). The resulting suspension was filtered through a 70 μm mesh sieve and then centrifuged at 1200 rpm for 8 min at room temperature. After the supernatant was discarded, the pellet was resuspended in complete DMEM/F-12 medium containing 10% FBS (P30-2602; PAN, Germany), 10 ng/mL human basic fibroblast growth factor (100-18B-100, PeproTech, Cranbury, NJ, USA) and 2 mM L-glutamine. The cells were then inoculated in a 60 mm dish and incubated at 37 °C with 5% CO_2_. Non-adherent cells were removed 48 h after initial plating and washed with PBS.

### 2.3. Immunofluorescence

Monolayer cultures of cattle ADSCs were fixed in 4% paraformaldehyde for 30 min and then washed 3 times with PBS. After that, the cattle ADSCs were fixed in 10% methanol for 10 min in −20 °C. Then, the cells were permeabilized with 0.25% TritonX-100 for 15 min and also washed 3 times in PBS. After being blocked with PBS containing 0.3% BSA (sigma, USA), the cells were incubated in PBS containing the following polyclonal antibodies overnight at 4 °C: mouse anti-CD29 (1:100; 610468, BD, Franklin Lakes, NJ, USA), mouse anti-CD44 (1:200; ab254530, Abcam, Waltham, MA, USA), rabbit anti-CD73 (1:100, bs-4834R, Bioss, Xiaotangshan, China), rabbit anti-CD90 (1:100; WL01236, Wanlei, China), rabbit anti-CD105 (1:200, WL02945, Wanlei, Guangdong, China), rabbit anti-Vimentin (1:100; bs-0756R, Bioss, China) and rabbit anti-CD34 (1:500; WL02529, Wanlei, China). The next day, the primary antibody was removed and the cells were washed 3 times with PBS. We then added FITC-conjugated (1:200, ZF-0308, Zhongshan Golden Bridge; Beijing, China) or PE-conjugated secondary antibodies (1:200, bs-0296G, Bioss, China) and incubated the samples at room temperature in the dark for 1 h. Nuclei were stained with 1 μL/mL DAPI for 15 min, and then washed 3 times with PBS. Images were acquired using a laser-scanning confocal microscope (Nikon TE-2000-E). As technical controls, PBS was used in place of primary antibodies. Ten randomly selected non-overlapping fields of view were observed and photographed.

### 2.4. Cell Culture and Treatment

Cattle ADSCs were cultured in DMEM/F12 medium with 10% fetal bovine serum and 1% penicillin/streptomycin in the incubator (37 °C, 5% CO_2_). Upon reaching 70–80% confluence, the cells were exposed to H_2_O_2_ for 24 h, as indicated. Curcumin (Cur) was applied to the cells while H_2_O_2_ was added in the medium. The pharmacological inhibitors 3-methyladenine (3-MA, 10 μM) were added 30 min before the cells were exposed to H_2_O_2_.

### 2.5. Cell Viability Analysis

The Cell Counting Kit-8 (CCK-8) assay was used to detect the cell viability. Briefly, cells were inoculated and incubated in 96-well plates for 24 h, then treated with different concentrations of curcumin for 24 h. Afterward, the cells in each well were treated according to the instructions of the CCK-8 kit (Beyotime Biotechnology, Shanghai, China). The survival rate of cells was calculated as a relative percentage of the untreated control.

### 2.6. Determination of ROS Production and MDA Activity

The 2′,7′-dichlorofluorescin diacetate (DCFH-DA) assay was employed to determine the intracellular ROS levels. Cattle ADSCs were inoculated in 96-well plates and protected with various concentrations of curcumin for 30 min; then, the cells were exposed to H_2_O_2_ for 12 h. Then, the bovine ADSCs were incubated with DCFH-DA (Beyotime Institute of Biotechnology, Shanghai, China) for 30 min at 37 °C in the dark. The ROS levels were determined by measuring the fluorescence intensity using a Multi-Mode Microplate Reader (PerkinElmer, Waltham, MA, USA) under the same exposure conditions. The results were presented as relative fold-changes compared with the normal controls.

For MDA detection, H_2_O_2_-stimulated Cattle ADSCs were harvested with ice-cold RIPA containing 1 mM of PMSF, and the BCA protein assay kit (Beyotime Biotechnology, Shanghai, China) was employed to determine the protein concentration. The MDA levels were then measured using the corresponding detection kit (Jiancheng, Nanjing, China) according to the manufacturer’s protocols.

### 2.7. Determination of SOD Activities

H_2_O_2_-stimulated Cattle ADSCs with different concentrations of curcumin were collected and centrifuged before harvesting the supernatant and detecting the SOD activity using the corresponding detection kits (Beyotime Biotechnology, Shanghai, China). In addition, the protein was determined as previously described. The results were presented as relative fold-changes compared with the normal controls.

### 2.8. TUNEL

H_2_O_2_-stimulated Cattle ADSCs with different concentrations of curcumin were fixed in 4% paraformaldehyde for 30 min and then washed 3 times with PBS. Apoptosis was measured using the TUNEL apoptosis detection kit (Beyotime Biotechnology, Shanghai, China).

### 2.9. Western-Blot

Cells were harvested, and the protein was quantified as previously described. Then, SDS/PAGE was used to separate the samples before transferring the samples onto PVDF membranes (ISEQ00010, Millipore, Germany). After blocking and washing, the membranes were incubated with primary antibodies against rabbit anti-P62 (A7758, ABclone, China) and rabbit anti-β-tublin (BC0170, ABGENT, Shanghai, China). They were then probed with secondary antibodies (goat anti-rabbit IgG; Abkine; A21020), as previously reported. The antibody–antigen complexes were determined using a chemiluminescence assay system. ImageJ software (1.53a) was employed to calculate the density of protein bands. β-tublin was selected as the internal standard.

### 2.10. Statistical Analysis

All data are shown as the mean ± SD from no fewer than three independent experiments. Statistical differences were evaluated by a two-tailed *t*-test or ANOVA, followed by the Student–Newman–Keuls test. *p* < 0.05 was considered to indicate a statistically significant difference.

## 3. Results

### 3.1. Isolation and Culture of Cattle Adipose Derived Stem Cells

Cattle ADSCs were isolated from subcutaneous adipose tissue of fetal cattle at 5 to 6 months. The Immunofluorescence of Cattle ADSCs surface markers at the fifth passages revealed the co-expression of CD29, CD44, CD73, CD90, CD105 and Vimenntin, but not CD34 (Supplementary Figure S1).

### 3.2. H_2_O_2_ Induce Oxidative Stress and Apoptosis of Cattle ADSCs

**Figure 1:**
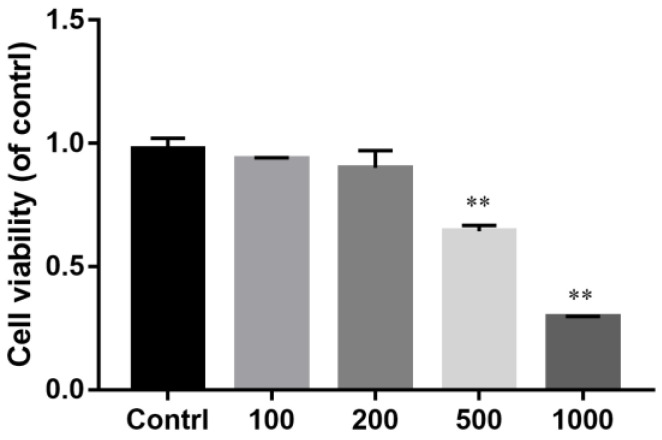
Effect of H_2_O_2_ on viability of cattle ADSCs. Cattle ADSCs were treated with different concentrations (0 μM, 100 μM, 200 μM and 500 μM) of H_2_O_2_ for 24 h and then assayed by the CCK-8 test. The data are expressed as the mean ± SD of three experiments. “**” means that the result is statistically significant compared with the control group (*p* < 0.01).

Firstly, cattle ADSCs (adipose-derived stem cells) were stimulated with different concentrations of H_2_O_2_ for 24 h. Then, the CCK-8 assay was used to detect the viability of the cattle ADSCs. The results revealed that there was no significant change in viability when the concentrations of H_2_O_2_ reached 100 mM and 200 mM (Figure 1). When the concentration of H_2_O_2_ reached 500 mM, the cell viability of the cattle ADSCs decreased significantly (*p* < 0.01) (Figure 1). When the concentration of H_2_O_2_ reached 1000 mM, the ADSCs had mostly died (Figure 1). Therefore, in follow-up experiments, 500 mM of H_2_O_2_ was used to induce oxidative stress and apoptosis in cattle ADSCs.

**Figure 2:**
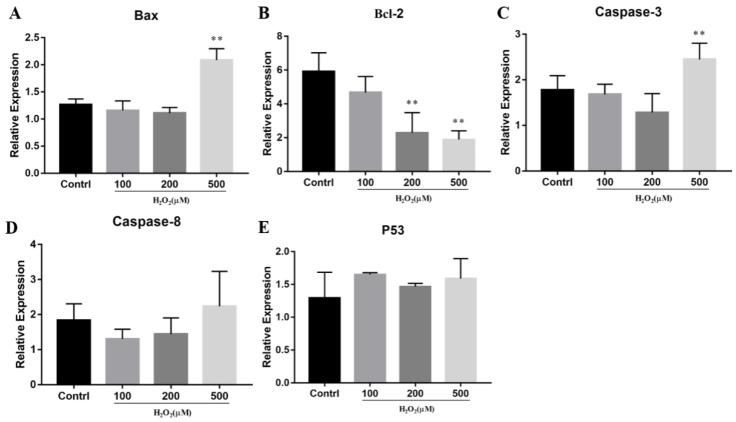
Effects of H_2_O_2_ on apoptosis marker genes. Cattle ADSCs were treated with different concentrations (0 μM, 100 μM, 200 μM and 500 μM) of H_2_O_2_ for 24 h. Then, the expression of apoptosis marker genes ((**A**–**E**): *Bax*, *Bcl-2*, *Caspase-3*, *Caspase-8* and *P53*) was detected by qPCR. “**”, *p* < 0.01.

Secondly, qPCR was used to detect the expression of apoptosis-related genes in the oxidative stress model of cattle adipose-derived stem cells (ADSCs). The expressions of *Bax*, *Bcl-2*, and *caspase-3* were significantly changed when the H_2_O_2_ concentration reached 500 mM (*p* < 0.05) (Figure 2), indicating that 500 mM of H_2_O_2_ can induce apoptosis in cattle ADSCs.

**Figure 3:**
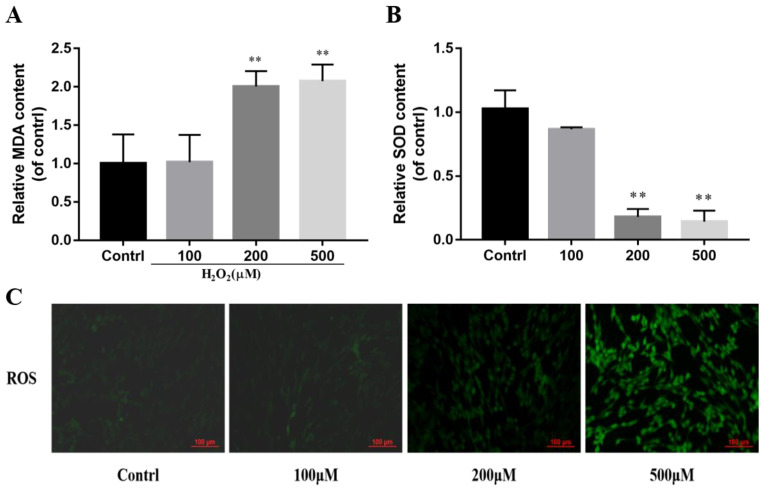
Effect of H_2_O_2_ on the generation of MDA (**A**), SOD activity (**B**), and generation of ROS (**C**) in cattle ADSCs. Cattle ADSCs were treated with different concentrations (1 μM, 10 μM, 50 μM and 100 μM) of H_2_O_2_ for 24 h. Then, the cells were collected and used for MDA (**A**) and SOD (**B**) and ROS (**C**) analysis (Bar = 100 μm). Note: “**” indicates extremely significant difference (*p* < 0.01).

Thirdly, this study also examined the changes in oxidative stress-related indicators of bovine ADSCs under oxidative stress models. As shown in Figure 3, compared with the blank group, when the concentration of H_2_O_2_ reached 200 μM and 500 μM, malondialdehyde (MDA) increased significantly (*p* < 0.01) (Figure 3A). As shown in Figure 3B, SOD activity decreased significantly (*p* < 0.01). As shown in Figure 3C, 500 μmol/L H_2_O_2_ increased the ROS content of bovine ADSCs.

### 3.3. Curcumin Improving Oxidative Stress and Apoptosis of Bovine ADSCs

**Figure 4:**
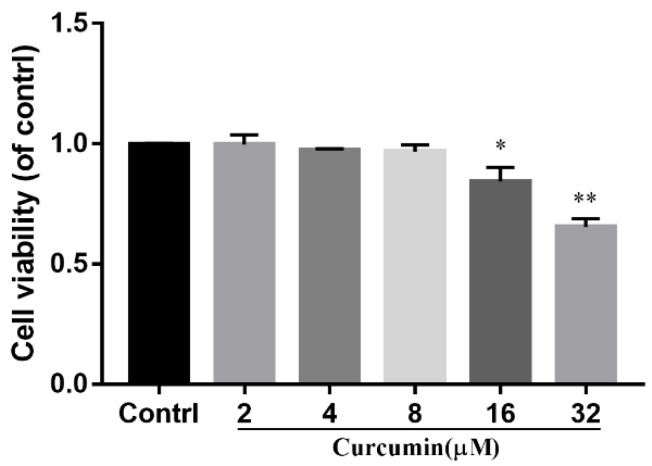
Effect of curcumin on viability of cattle ADSCs. Cattle ADSCs were incubated with different concentrations (0 μM, 2 μM, 4 μM, 8 μM, 16 μM and 32 μM) of curcumin for 24 h (DMSO was used as the solvent for curcumin in all treatment groups, including the control group treated with DMSO alone) and then assayed by CCK-8. The data are expressed as the mean ± SD of three experiments. “*”, *p* < 0.05, “**”, *p* < 0.01.

To determine the optimal concentration of curcumin, the effects of different concentrations of curcumin on the viability of bovine ADSCs were analyzed. Bovine ADSCs were treated with different concentrations of curcumin for 24 h, and the effects on the viability of bovine ADSCs were detected through CCK-8 experiments. As shown in Figure 4, compared with the blank group, when the concentration of curcumin added was less than 8 μM, there was no significant effect on cell viability. When curcumin reached 16 μM, the cell survival rate decreased significantly (*p* < 0.05).

**Figure 5:**
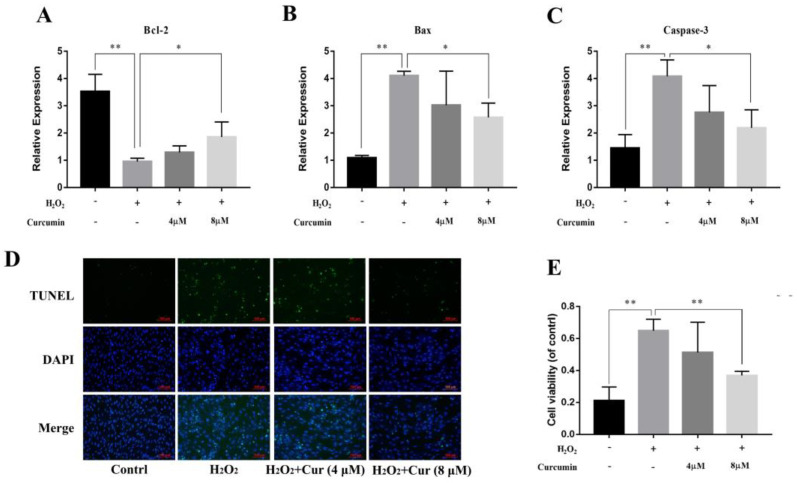
Effect of curcumin on apoptosis of cattle ADSCs. Cattle ADSCs were treated with different concentrations of curcumin (0 μM, 4 μM and 8 μM; DMSO was used as the solvent for curcumin in all treatment groups, including the control group treated with DMSO alone) under the H_2_O_2_ stimulation for 24 h, after which cells were collected and used for qPCR and TUNEL analysis. (**A**–**C**) The expression of apoptosis marker genes: *Bcl*–*2*, *Bax* and *Caspase*–*3*. (**D**) Apoptotic cells in heart sections were labeled by TUNEL staining (Bar = 100 μm). The numbers of TUNEL–positive cells were quantified in the graph (**E**). The data are expressed as the mean ± SD of three experiments. “*”, *p* < 0.05, “**”, *p* < 0.01.

Oxidative stress often leads to apoptosis, and previous experiments have demonstrated that bovine ADSCs undergo apoptosis under H_2_O_2_ treatment. Additionally, this chapter’s research also proves that curcumin has a certain mitigating effect on oxidative stress in bovine ADSCs. Therefore, this study further investigated the impact of curcumin on apoptosis induced by oxidative stress in bovine ADSCs. Firstly, RT-qPCR was used to detect apoptosis-related genes. As shown in Figure 5, curcumin can significantly inhibit the decrease in Bcl-2 gene expression caused by H_2_O_2_ (*p* < 0.01) and can significantly inhibit the increase in Bax and Caspase-3 gene expression induced by H_2_O_2_ (*p* < 0.05) (Figure 5A–C).

Furthermore, this study also verified the anti-apoptotic effect of curcumin through the TUNEL assay. By calculating the apoptosis rate (Figure 5D,E), it was found that curcumin can alleviate apoptosis in bovine ADSCs induced by H_2_O_2_.

**Figure 6:**
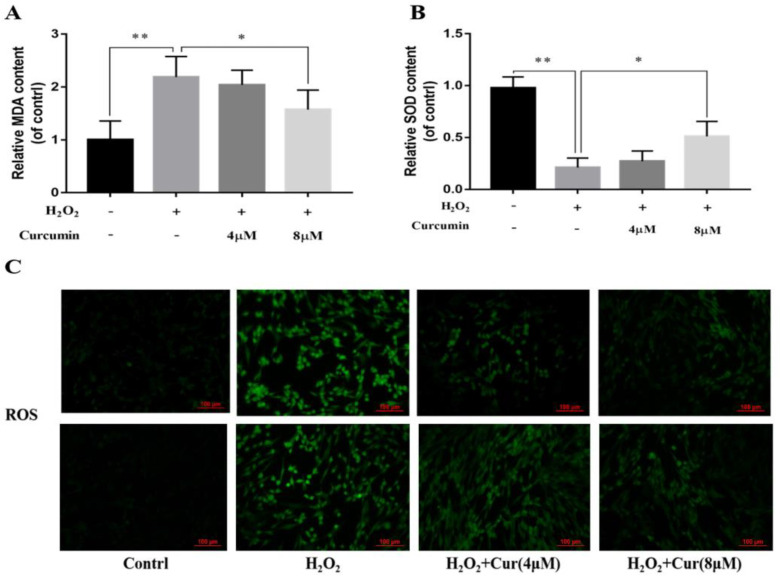
Effect of curcumin on the generation of MDA (**A**), SOD (**B**) and ROS (**C**) in cattle ADSCs. Cattle ADSCs were treated with different concentrations of curcumin (0, 4 and 8 μM; DMSO was used as the solvent for curcumin in all treatment groups, including the control and H_2_O_2_ group) under the H_2_O_2_ stimulation for 24 h, after which cells were collected and used for MDA (**A**) and SOD (**B**) and ROS (**C**) analysis (Bar = 100 μm). “*”, *p* < 0.05, “**”, *p* < 0.01.

Previous studies have reported that curcumin has protective effects against oxidative damage in different cell lines. This study explored the effects of curcumin on oxidative stress models of bovine ADSCs. As shown in Figure 6, under oxidative stress, when the concentration of curcumin reached 8 μM, it could significantly reduce the MDA content (*p* < 0.05) (Figure 6A) and also maintain SOD activity (*p* < 0.05) (Figure 6B). In addition, curcumin at concentrations of 4 μM and 8 μM could alleviate the increase in the ROS content caused by oxidative stress in bovine ADSCs (Figure 6C).

### 3.4. 3-MA Inhibits Curcumin-Induced Autophagy and Affects Its Anti-Apoptotic and Antioxidant Effects

Studies have reported that curcumin can induce autophagy to help cells survive various stress responses. Therefore, this study further explores whether curcumin protects bovine ADSCs from oxidative stress and apoptosis by inducing autophagy. 3-MA is often used as an autophagy inhibitor due to its ability to effectively suppress autophagy. Consequently, this study employed a co-treatment of curcumin and 3-MA (10 mM) on an oxidative stress model of bovine ADSCs to investigate whether curcumin can continue to exert its antioxidant and anti-apoptotic effects after autophagy inhibition.

**Figure 7:**
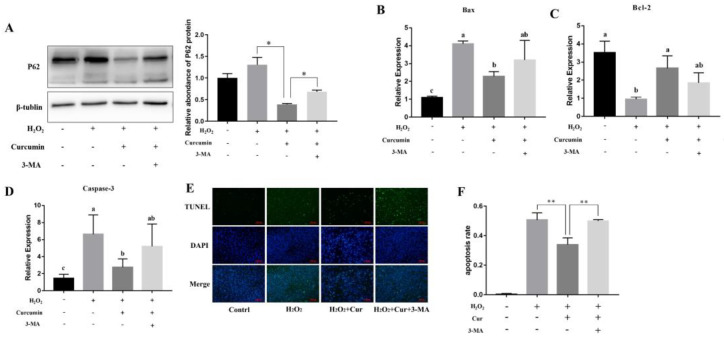
Effect of 3–MA on the antiapoptotic effect of curcumin. Bovine ADSCs (adipose-derived stem cells) were co-treated with H2O2, curcumin (DMSO was used as the solvent for curcumin in all treatment groups), and 3–MA. (**A**) The expression levels of P62 proteins were detected by Western Blot. (**B**–**D**) The expression levels of Bax (**B**), Bcl–2 (**C**) and Caspase–3 (**D**) were detected by qPCR. (**E**) The effect of 3–MA on the antiapoptotic effect of curcumin was detected by TUNEL (Bar = 100 μm). (**F**) Statistical analysis on (**E**). Note: “*”, *p* < 0.05, “**”, *p* < 0.01; “a”, “b” and “c” are used to indicate whether the differences between different groups are significant. Where there is a same mark letter, the difference is not significant (*p* > 0.05), and where there are different mark letters, the difference is significant (*p* < 0.05).

First, Western blot analysis was used to detect the autophagy-related proteins LC3-II and P62. As shown in Figure 7A, under oxidative stress conditions, curcumin promoted an increase in LC3-II expression and a decrease in P62 expression, indicating that it can induce autophagy in bovine ADSCs. Additionally, 3-MA was able to inhibit curcumin-induced autophagy.

**Figure 8:**
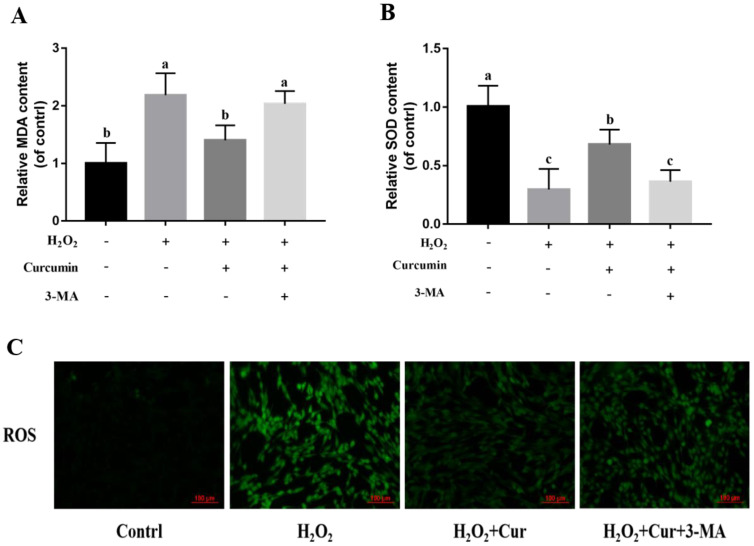
Bovine ADSCs (adipose-derived stem cells) are co-treated with H_2_O_2_, curcumin (DMSO was used as the solvent for curcumin in all treatment groups), and 3–MA. (**A**) Effects of 3–MA and curcumin on the generation of MDA, (**B**) effects of 3-MA and curcumin on the generation of SOD, and (**C**) effects of 3–MA and curcumin on the generation of ROS. Note: “a”, “b” and “c” are used to indicate whether the differences between different groups are significant. Where there is a same mark letter, the difference is not significant (*p* > 0.05), and where there are different mark letters, the difference is significant (*p* < 0.05).

Secondly, this study explored whether curcumin exerts its antioxidant effects through autophagy. Compared to the H_2_O_2_ group, the curcumin group reduced the expression of Bax and Caspase-3 genes (*p* < 0.05) (Figure 7B,D) and upregulated the expression of the Bcl-2 gene (*p* < 0.05) (Figure 7C) in bovine ADSCs. However, when 3-MA was co-treated with curcumin, there were no significant differences in the expression of these genes compared to the H_2_O_2_ group (*p* > 0.05), indicating that the anti-apoptotic effect of curcumin under oxidative stress was weakened by the addition of 3-MA (Figure 7B–D). 3-MA inhibited the anti-apoptotic effect of curcumin, leading to an increase in the apoptosis rate of bovine ADSCs (*p* < 0.05). Compared to the H_2_O_2_ group, the curcumin group had a reduced MDA content (*p* < 0.05) (Figure 8A) and increased SOD activity (*p* < 0.05) (Figure 8B). However, when 3-MA was co-treated with curcumin, there were no significant differences in the MDA content and SOD activity compared to the H_2_O_2_ group (*p* > 0.05). Additionally, compared to the curcumin-treated group, the co-treatment of 3-MA and curcumin resulted in an increase in the ROS content (Figure 8C), indicating that the antioxidant effect of curcumin under oxidative stress was weakened by the addition of 3-MA.

## 4. Discussion

The balance between oxidation and antioxidant plays a pivotal role in maintaining homeostasis in organisms. An imbalance between the two can lead to oxidative stress within the body, representing the adverse effects of free radicals in the body. When an animal’s body is under oxidative stress, various indicators become unbalanced, causing oxidative damage. Furthermore, oxidative stress is considered a crucial factor contributing to aging and disease. Stem cells play an essential role in tissue repair, self-renewal, and other functions of the body, and their functional decline often has adverse effects on the animal’s body. Therefore, this study uses H_2_O_2_ to induce oxidative stress and apoptosis in bovine adipose-derived stem cells (ADSCs) and explores the ameliorative effects of active substances such as curcumin on oxidative stress and apoptosis in bovine ADSCs.

H_2_O_2_ is a common potent oxidant frequently used in cellular oxidative stress modeling (Kaczara et al. 2010). However, the modeling methods vary significantly among different cell types. Based on previous research on H_2_O_2_-induced oxidative stress modeling, 24 h was selected as the treatment duration for H_2_O_2_ [28,29]. To determine the appropriate treatment concentration, cell viability was assessed using the CCK-8 assay. It was found that when the H_2_O_2_ concentration reached 500 μM, cell viability decreased to approximately 60%, indicating that the cells’ growth state was poor. When the H_2_O_2_ concentration reached 1000 μM, the cell viability continued to decline significantly, with most cells dying, making it unsuitable for subsequent research. Therefore, 500 μM of H_2_O_2_ was chosen to induce oxidative stress in bovine ADSCs for 24 h to establish the oxidative stress model. To verify the reliability of the model, various experiments were conducted. It was observed that 500 μM of H_2_O_2_ inhibited the activity of superoxide dismutase (SOD) in bovine ADSCs and significantly increased the levels of reactive oxygen species (ROS) and malondialdehyde (MDA). Finally, RT-qPCR revealed that H_2_O_2_ could also promote apoptosis in bovine ADSCs. In summary, these results demonstrate that treatment with 500 μM of H_2_O_2_ for 24 h can effectively establish an oxidative stress model in bovine ADSCs.

**Figure 9:**
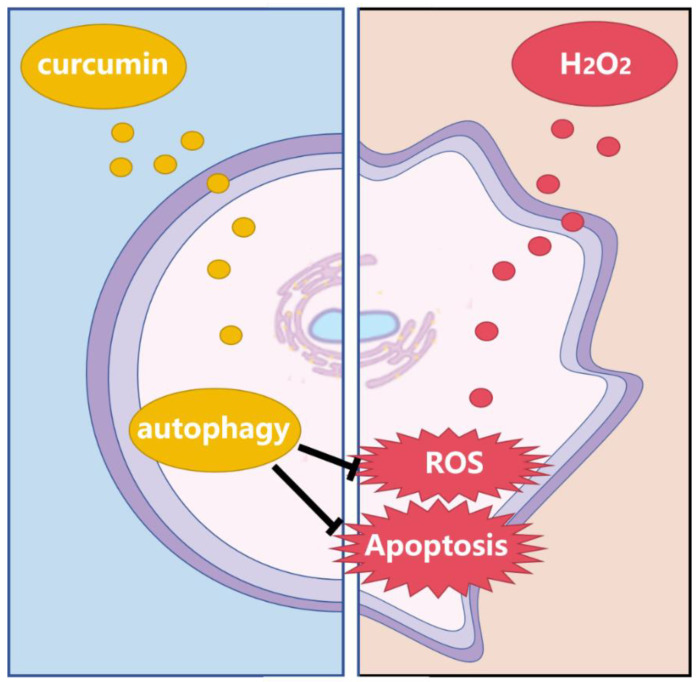
Molecular mechanism diagram of curcumin inhibiting oxidative stress and apoptosis of bovine ADSCs.

In previous studies, it was found that H_2_O_2_ elevates cellular reactive oxygen species (ROS) through various mechanisms such as direct oxidation, the Fenton reaction, and the modulation of cellular signaling pathways. Excessive or sustained increases in ROS can induce oxidative stress in cells. ROS can trigger cell apoptosis through several pathways: 1. ROS can directly act on DNA, causing base modifications and DNA strand breaks. If the DNA damage is severe and unrepairable, the cell will initiate the apoptotic program. In this process, the *p53* gene plays a pivotal role. *P53* is a tumor suppressor gene that, when activated by DNA damage, promotes cell apoptosis [30,31]. 2. ROS can attack mitochondrial membranes, leading to mitochondrial dysfunction and reduced ATP production. When ATP levels drop to a certain threshold, cells are unable to maintain normal physiological functions and subsequently initiate the apoptotic program [32,33]. Furthermore, mitochondrial DNA (mtDNA) is highly sensitive to oxidative stress and prone to damage that results in mitochondrial dysfunction, further promoting cell apoptosis [34]. 3. ROS can also attack unsaturated fatty acids on the cell membrane, directly damaging the membrane through lipid peroxidation [35]. This membrane damage alters membrane permeability and stability, leading to an increased influx of ions such as Ca^2+^ and the activation of apoptotic signaling pathways [36]. 4. Oxidative stress can also activate nuclear transcription factors such as NF-κB and AP-1. These transcription factors play crucial roles in the process of cell apoptosis by accelerating the expression of apoptosis-related genes, thereby inducing cell apoptosis [37]. We have shown the process of H_2_O_2_-induced ADSCs in Figure 9.

Curcumin, a plant polyphenol, has been extensively studied for its medicinal value. Through continuous exploration of its structural and functional properties, researchers have discovered its significant roles in animal inflammation, oxidative stress, autophagy, and other processes. However, relatively few studies have focused on its effects on bovine adipose-derived stem cells (ADSCs). This study aimed to investigate the impact of curcumin on oxidative stress and apoptosis in bovine ADSCs. Specifically, it prevents a significant decline in SOD activity in bovine ADSCs due to oxidative stress and significantly reduces intracellular levels of MDA and ROS. Additionally, curcumin was found to significantly inhibit H_2_O_2_-induced apoptosis.

Previous research has reported that curcumin can enhance cell survival and exert anti-apoptotic effects by inducing autophagy. For instance, curcumin inhibits H9c2 cell apoptosis through autophagy induction [38]. It also promotes autophagy in hepatocytes, inhibiting epithelial–mesenchymal transition and thereby suppressing the development and progression of liver fibrosis [19]. Furthermore, curcumin activates autophagy to regulate inflammatory responses and oxidative stress, effectively preventing hepatic steatosis in experimental non-alcoholic fatty liver disease rat models [20]. Autophagy is a self-protection mechanism where damaged proteins and organelles are transported to lysosomes for degradation in response to external stimuli such as oxidative stress, thereby maintaining homeostasis [39]. Therefore, this study further explored whether curcumin exerts its antioxidant and anti-apoptotic effects in bovine ADSCs by promoting autophagy (Figure 9).

Using Western blot analysis, the study found that curcumin promoted the expression of LC3-II and P62 proteins in bovine ADSCs under oxidative stress conditions, suggesting that curcumin can induce autophagy in bovine ADSCs under oxidative stress. Additionally, 3-MA, a commonly used autophagy inhibitor, was employed to verify its inhibitory effect on curcumin-induced autophagy in bovine ADSCs. The Western blot results confirmed that 3-MA inhibited curcumin-induced autophagy, as evidenced by the altered LC3-II and P62 protein levels. Further experiments demonstrated that 3-MA also attenuated the antioxidant and anti-apoptotic effects of curcumin, indicating that curcumin’s protective effects are mediated, at least in part, by autophagy.

Studies have shown that curcumin can induce autophagy in chondrocytes via the ERK1/2 signaling pathway and inhibit IL-1β-induced chondrocyte apoptosis through autophagy [18]. These findings are consistent with the results of the present study.

Despite the beneficial effects of curcumin on animal health, the issue of low curcumin bioavailability indeed exists, limiting its application in clinical treatment and health supplements [27]. Firstly, curcumin has low solubility in water, making it difficult for the human body to effectively absorb it. Secondly, it has poor stability; curcumin is less stable in strong acid or strong alkali environments and is prone to degradation, resulting in most of it being converted into other substances in the stomach. Lastly, curcumin is metabolized rapidly in the body, with most of it being quickly broken down after ingestion, further reducing its bioavailability. Therefore, many scholars are striving to improve curcumin’s bioavailability. Firstly, its physical form can be altered, preparing it into nanoparticles, for example, to enhance its stability by improving its solubility in water [40]. Secondly, combining curcumin with other ingredients (such as black pepper) can enhance its utilization [27]. These methods are used to improve the bioavailability of curcumin.

In conclusion, this study revealed that curcumin may inhibit oxidative stress responses in bovine ADSCs by inducing autophagy, demonstrating that a certain dose of curcumin exerts antioxidant effects that could be applied in animal husbandry to mitigate oxidative stress, which is of great significance for livestock health and the development of the animal husbandry industry. The molecular mechanism involved in the inhibition of oxidative stress and apoptosis by curcumin via the promotion of autophagy in bovine ADSCs is shown in Figure 9.

## Data Availability

Dataset available on request from the authors. The raw data supporting the conclusions of this article will be made available by the authors on request.

## Supplementary Materials

The following supporting information can be downloaded at: https://www.mdpi.com/article/10.3390/ani14233421/s1, Figure S1: Immunofluorescence identification of cattle ADSC surface marker proteins (CD29, CD44, CD73, CD90, CD105, Vimentin and CD34. NC: No primary antibody was added).

## References

[1] Miller J.K., Brzezinska S.E., Madsen F.C. (1993). Oxidative stress, antioxidants, and animal function. J. Dairy Sci..

[2] Rita T., Simone B., Vincenzo C. (2021). Improving Curcumin Bioavailability: Current strategies and future perspectives. Pharmaceutics.

[3] Qin Y., Ge G., Yang P., Wang L., Qiao Y., Pan G., Yang H., Bai J., Cui W., Geng D. (2023). An update on adipose-derived stem cells for regenerative medicine: Where challenge meets opportunity. Adv. Sci..

[4] Bacakova L., Zarubova J., Travnickova M., Musilkova J., Pajorova J., Slepicka P., Kasalkova N.S., Svorcik V., Kolska Z., Motarjemi H. (2018). Stem cells: Their source, potency and use in regenerative therapies with focus on adipose-derived stem cells—A review. Biotechnol. Adv..

[5] Lee S., Chae D.S., Song B.W., Lim S., Kim S.W., Kim I.K., Hwang K.C. (2021). ADSC-based cell therapies for musculoskeletal disorders: A review of recent clinical trials. Int. J. Mol. Sci..

[6] Mansouri K., Rasoulpoor S., Daneshkhah A., Abolfathi S., Salari N., Mohammadi M., Rasoulpoor S., Shabani S. (2020). Clinical effects of curcumin in enhancing cancer therapy: A systematic review. BMC Cancer.

[7] Abadi A.J., Mirzaei S., Mahabady M.K., Hashemi F., Zabolian A., Hashemi F., Raee P., Aghamiri S., Ashrafizadeh M., Aref A.R. (2022). Curcumin and its derivatives in cancer therapy: Potentiating antitumor activity of cisplatin and reducing side effects. Phytother. Res..

[8] Nawa A., Ma L., Wang R., Nan Y., Li W., Wang Q., Jin F. (2019). Effect of curcumin supplementation on TLR4 mediated non-specific immune responses in liver of laying hens under high-temperature conditions. J. Therm. Biol..

[9] Lin X., Bai D., Wei Z., Zhang Y., Huang Y., Deng H., Huang X. (2019). Curcumin attenuates oxidative stress in RAW264.7 cells by increasing the activity of antioxidant enzymes and activating the Nrf2-Keap1 pathway. PLoS ONE.

[10] Betbeder D., Lipka E., Howsam M., Carpentier R. (2015). Evolution of availability of curcumin inside poly-lactic-co-glycolic acid nanoparticles: Impact on antioxidant and antinitrosant properties. Int. J. Nanomed..

[11] Chopra H., Dey P.S., Das D., Bhattacharya T., Shah M., Mubin S., Maishu S.P., Akter R., Rahman M.H., Karthika C. (2021). Curcumin nanoparticles as promising therapeutic agents for drug targets. Molecules.

[12] Kong Z.L., Kuo H.P., Johnson A., Wu L.C., Chang K.L.B. (2019). Curcumin-loaded mesoporous silica nanoparticles markedly enhanced cytotoxicity in hepatocellular carcinoma cells. Int. J. Mol. Sci..

[13] Ceccariglia S., Cargnoni A., Silini A.R., Parolini O. (2020). Autophagy: A potential key contributor to the therapeutic action of mesenchymal stem cells. Autophagy.

[14] Ceccariglia S., Alvino A., Del Fà A., Parolini O., Michetti F., Gangitano C. (2019). Autophagy is Activated In Vivo during Trimethyltin-induced apoptotic neurodegeneration: A study in the rat hippocampus. Int. J. Mol. Sci..

[15] Perrone L., Squillaro T., Napolitano F., Terracciano C., Sampaolo S., Melone M.B. (2019). The autophagy signaling pathway: A potential multifunctional therapeutic target of curcumin in neurological and neuromuscular diseases. Nutrients.

[16] Wang B., Wang Y., Zhang J., Hu C., Jiang J., Li Y., Peng Z. (2023). ROS-induced lipid peroxidation modulates cell death outcome: Mechanisms behind apoptosis, autophagy, and ferroptosis. Arch. Toxicol..

[17] Jain A., Lamark T., Sjøttem E., Larsen K.B., Awuh J.A., Øvervatn A., McMahon M., Hayes J.D., Johansen T. (2010). p62/SQSTM1 is a target gene for transcription factor NRF2 and creates a positive feedback loop by inducing antioxidant response element-driven gene transcription. J. Biol. Chem..

[18] Li L., Tan J., Miao Y., Lei P., Zhang Q. (2015). ROS and autophagy: Interactions and molecular regulatory mechanisms. Cell. Mol. Neurobiol..

[19] Zhang F., Zhang Z., Chen L., Kong D., Zhang X., Lu C., Lu Y., Zheng S. (2014). Curcumin attenuates angiogenesis in liver fibrosis and inhibits angiogenic properties of hepatic stellate cells. J. Cell. Mol. Med..

[20] White C.M., Lee J.Y. (2019). The impact of turmeric or its curcumin extract on nonalcoholic fatty liver disease: A systematic review of clinical trials. Pharm. Pract..

[21] Ruan D., Wang W.C., Lin C.X., Fouad A.M., Chen W., Xia W.G., Wang S., Luo X., Zhang W.H., Yan S.J. (2018). Effects of curcumin on performance, antioxidation, intestinal barrier and mitochondrial function in ducks fed corn contaminated with ochratoxin A. Animal.

[22] Hao F., Kang J., Cao Y. (2015). Curcumin attenuates palmitate-induced apoptosis in MIN6 pancreatic β-cells through PI3K/Akt/FoxO1 and mitochondrial survival pathways. Apoptosis.

[23] Hung Y.L., Fang S.H., Wang S.C., Cheng W.C., Liu P.L., Su C.C., Chen C.S., Huang M.Y., Hua K.F., Shen K.H. (2017). Corylin protects LPS-induced sepsis and attenuates LPS-induced inflammatory response. Sci. Rep..

[24] Gan Z., Wei W., Li Y., Wu J., Zhao Y., Zhang X., Wang T., Zhong X. (2019). Curcumin and resveratrol regulate intestinal bacteria and alleviate intestinal inflammation in weaned piglets. Molecules.

[25] Joung H.J., Choi M.J., Kim J.T., Park S.H., Park H.J., Shin G.H. (2016). Development of food grade curcumin nanoemulsion and its potential application to food beverage system: Antioxidant property and in vitro digestion. J. Food Sci..

[26] Aniket L., Roch C.Y., Cheng C.C., Je R.L., Kuan C.C. (2018). Protective and detoxifying effects conferred by dietary selenium and curcumin against afb1-mediated toxicity in livestock: A review. Toxins.

[27] Strimpakos A.S., Sharma R.A. (2008). Curcumin: Preventive and therapeutic properties in laboratory studies and clinical trials. Antioxid. Redox Signal..

[28] Patrycja K., Tadeusz S., Janice M. (2010). Dynamics of H_2_O_2_ availability to ARPE-19 cultures in models of oxidative stress. Free Radic. Biol. Med..

[29] Ren R., Chen S.D., Fan J., Zhang G., Li J.B. (2018). MiRNA-138 regulates MLK3/JNK/MAPK pathway to protect BV-2 cells from H2O2-induced apoptosis. Bratisl. Med. J..

[30] Srinivas U.S., Tan B.W.Q., Vellayappan B.A., Jeyasekharan A.D. (2019). ROS and the DNA damage response in cancer. Redox Biol..

[31] Yu G., Luo H., Zhang N., Wang Y., Li Y., Huang H., Liu Y., Hu Y., Liu H., Zhang J. (2019). Loss of p53 sensitizes cells to palmitic acid-induced apoptosis by reactive oxygen species accumulation. Int. J. Mol. Sci..

[32] Zheng W.L., Wang B.J., Wang L., Shan Y.P., Zou H., Song R.L., Wang T., Gu J.H., Yuan Y., Liu X.Z. (2018). ROS-Mediated Cell Cycle Arrest and Apoptosis Induced by Zearalenone in Mouse Sertoli Cells via ER Stress and the ATP/AMPK Pathway. Toxins.

[33] Winitchaikul T., Sawong S., Surangkul D., Srikummool M., Somran J., Pekthong D., Kamonlakorn K., Nangngam P., Parhira S., Srisawang P. (2021). Calotropis gigantea stem bark extract induced apoptosis related to ROS and ATP production in colon cancer cells. PLoS ONE.

[34] Luo Z., Xu X., Sho T., Zhang J., Xu W., Yao J., Xu J. (2019). ROS-induced autophagy regulates porcine trophectoderm cell apoptosis, proliferation, and differentiation. Am. J. Physiol. Cell Physiol..

[35] Liu T., Sun L., Zhang Y., Wang Y., Zheng J. (2022). Imbalanced GSH/ROS and sequential cell death. J. Biochem. Mol. Toxicol..

[36] Paillamanque J., Madrid C., Carmona E.M., Osses N., Moreno R.D., Oresti G.M., Pino J.A., Reyes J.G. (2016). Effects of fatty acids on intracellular [Ca^2+^], mitochondrial uncoupling and apoptosis in rat pachytene spermatocytes and round spermatids. PLoS ONE.

[37] Hayes J.D., Dinkova-Kostova A.T., Tew K.D. (2020). Oxidative stress in cancer. Cancer Cell.

[38] Qing Y., Zhi Q.K., Shu G. (2018). Curcumin protects against diabetic cardiomyopathy by promoting autophagy and alleviating apoptosis. J. Mol. Cell. Cardiol..

[39] Choi Y., Bowman J., Jung J. (2018). Autophagy during viral infection—A double-edged sword. Nat. Rev. Microbiol..

[40] Yavarpour-Bali H., Ghasemi-Kasman M., Pirzadeh M. (2019). Curcumin-loaded nanoparticles: A novel therapeutic strategy in treatment of central nervous system disorders. Int. J. Nanomed..

