# Supplementary material for: Curcumin Inhibits Oxidative Stress and Apoptosis Induced by H2O2 in Bovine Adipose-Derived Stem Cells (bADSCs)
Source: Animals (Basel). 2024 Nov 26;14(23):3421. doi: 10.3390/ani14233421 (PMC11640669; doi:10.3390/ani14233421)
Supplement: Supplementary file 1 [file animals-14-03421-s001.zip › animals-3219447-supplementary.pdf]

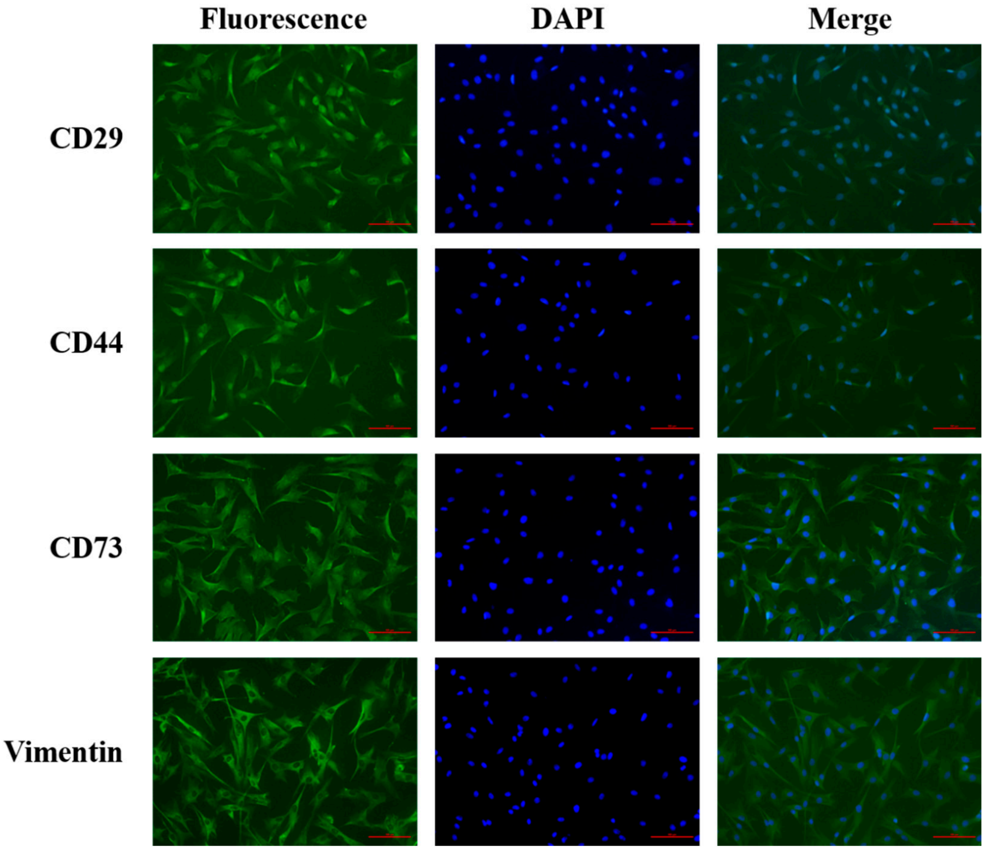

Supplementary Figure S1: Immunofluorescence identification of cattle ADSC surface marker proteins (scale bar=100μm)
